# A Novel MicroRNA-132-Surtuin-1 Axis Underlies Aberrant B-cell Cytokine Regulation in Patients with Relapsing-Remitting Multiple Sclerosis

**DOI:** 10.1371/journal.pone.0105421

**Published:** 2014-08-19

**Authors:** Yusei Miyazaki, Rui Li, Ayman Rezk, Hétoum Misirliyan, Craig Moore, Nasr Farooqi, Mayra Solis, Lorna Galleguillos Goiry, Omar de Faria Junior, Van Duc Dang, David Colman, Ajit Singh Dhaunchak, Jack Antel, Jennifer Gommerman, Alexandre Prat, Simon Fillatreau, Amit Bar-Or, on behalf of the CIHR/MSSC New Emerging Team Grant in Clinical Autoimmunity and the MSSRF Canadian B cells in MS Team

**Affiliations:** 1 Neuroimmunology Unit and Department of Neurology and Neurosurgery, Montreal Neurological Institute and Hospital, McGill University, Montreal, Quebec, Canada; 2 Clinical Research Unit, Montreal Neurological Institute and Hospital, McGill University, Montreal, Quebec, Canada; 3 Deutsches Rheuma-Forschungszentrum, Leibniz Institute, Berlin, Germany; 4 Department of Immunology, University of Toronto, Toronto, Ontario, Canada; 5 Neuroimmunology Research Unit, Centre de Recherche du Centre Hospitalier de l'Université de Montréal, Montreal, Quebec, Canada; 6 Experimental Therapeutics Program, Montreal Neurological Institute and Hospital, McGill University, Montreal, Quebec, Canada; University of British Columbia, Canada

## Abstract

Clinical trial results demonstrating that B-cell depletion substantially reduces new relapses in patients with multiple sclerosis (MS) have established that B cells play a role in the pathophysiology of MS relapses. The same treatment appears not to impact antibodies directed against the central nervous system, which underscores the contribution of antibody-independent functions of B cells to disease activity. One mechanism by which B cells are now thought to contribute to MS activity is by over-activating T cells, including through aberrant expression of B cell pro-inflammatory cytokines. However, the mechanisms underlying the observed B cell cytokine dysregulation in MS remain unknown. We hypothesized that aberrant expression of particular microRNAs might be involved in the dysregulated pro-inflammatory cytokine responses of B cells of patients with MS. Through screening candidate microRNAs in activated B cells of MS patients and matched healthy subjects, we discovered that abnormally increased secretion of lymphotoxin and tumor necrosis factor α by MS B cells is associated with abnormally increased expression of miR-132. Over-expression of miR-132 in normal B cells significantly enhanced their production of lymphotoxin and tumor necrosis factor α. The over-expression of miR-132 also suppressed the miR-132 target, sirtuin-1. We confirmed that pharmacological inhibition of sirtuin-1 in normal B cells induces exaggerated lymphotoxin and tumor necrosis factor α production, while the abnormal production of these cytokines by MS B cells can be normalized by resveratrol, a sirtuin-1 activator. These results define a novel miR-132-sirtuin-1 axis that controls pro-inflammatory cytokine secretion by human B cells, and demonstrate that a dysregulation of this axis underlies abnormal pro-inflammatory B cell cytokine responses in patients with MS.

## Introduction

Though traditionally viewed as a T cell-mediated disease, the demonstration that selective B-cell depletion in patients with multiple sclerosis (MS) leads to substantial reductions in the development of new focal brain lesions and clinical relapses [1]–[3], establishes an important role for B cells in mediating disease activity. The benefit of B-cell depletion in MS appears to occur without significantly impacting central nervous system (CNS)-autoreactive antibodies [2], indicating that the contribution of B-cells to MS relapses relates, at least in part, to antibody-independent functions of B-cells.

Normal B cells are now recognized to have the capacity to modulate T-cell responses through a number of antibody-independent mechanisms, including the expression of pro- or anti-inflammatory B cell cytokines [4]. Abnormalities in these B cell cytokine responses, resulting in exaggerated activation of T cells (or failure to properly regulate them), are thought to be relevant to how B cells contribute to new MS relapses [5], [6]. This concept is supported by observations in the commonly used experimental autoimmune encephalomyelitis (EAE) model of MS, where it has been shown that B cell-derived interleukin (IL)-6 and IL-10 can respectively enhance or suppress EAE disease activity [7]–[10]. We and subsequently others have shown that B cells of MS patients exhibit a defect in IL-10 production, suggesting a reduction of B cell-mediated immune regulation in MS [5], [11]. However, a deficiency in regulatory B cell function would not explain the benefit of B cell depletion (since removing an already defective cell would not be expected to make such a difference), hence there must be some abnormal pro-inflammatory B cell property in MS that is removed with the therapy. In this context, we have previously shown that activated MS B cells secrete abnormally high levels of the pro-inflammatory cytokines lymphotoxin (LT), tumor necrosis factor (TNF)α and IL-6, compared to B cells of healthy subjects (HS) [5], [11], and we have linked these abnormal B cell cytokine responses with abnormal (T helper) Th1 and Th17 T cell responses in both MS and EAE. The beneficial effects of B cell depletion therapy might therefore be related to an ablation of B cells producing pro-inflammatory cytokines in patients already deprived of effective B cell-mediated immune regulation. Accordingly, it might be possible to improve therapeutic B cell targeting in MS by developing novel tools that selectively inhibit their production of pro-inflammatory mediators, while preserving (or improving) their regulatory activities. To date, however, the molecular mechanism/s underlying abnormal B cell cytokine regulation in MS have not been elucidated. Indeed, relatively little is known about mechanisms that regulate normal B cell effector cytokine responses.

Micro (mi)RNAs are short single-strand oligonucleotides that regulate gene expression at a post-transcriptional level, and subsequently influence multiple biological processes [12]. Several studies have documented abnormal miRNA profiles in peripheral blood immune cells as well as CNS glial cells of patients with MS, compared to controls [13], [14], raising the possibility that such altered miRNA expression might be responsible for immunological features associated with MS pathogenesis. For instance, abnormal miRNA expression profiles have been linked with enhanced pathogenic functions of T cells in MS [15]–[17]. However, it is so far unknown whether any of the abnormal functions of B cells identified in MS are due to defects in miRNA expression. miRNA are known to be important for normal B cell physiology, including their development and maturation [18]–[22]. A recent study described, without assessing functional implications, an altered expression profile of miRNA in MS B cells [23]. We hypothesized that aberrant expression of particular miRNAs might be involved in the dysregulated pro-inflammatory cytokine responses of B cells of patients with MS.

## Materials and Methods

### Ethics statement

This study is approved by the ethics review board of the Montreal Neurological Institute and Hospital, and all subjects provided written informed consent according to the approval.

### Subjects

Venous blood samples were obtained from well-characterized patients with relapsing-remitting MS [24] (n = 19; average age: 45.68±2.24; female:male = 17∶2) and from demographically similar healthy subjects (HS; n = 19; average age: 44.42±2.44; female:male = 17∶2). All MS patients were untreated at time of blood draws, including no immune modulating treatment within at least 6 months and no steroid exposure within at least 90 days. None of the patients exhibited clinical features of active relapse for at least 3 months prior to the blood draws.

### B cell isolation and culture

Following Ficoll gradient (GE Healthcare) isolation of peripheral blood mononuclear cells (PBMC) from venous blood, total CD19^+^ B cells were positively isolated using CD19 microbeads (Miltenyi Biotec) based on our established protocols [5]–[7]. Purity confirmation was routinely ascertained as ≥98% using flow cytometry (Figure S1). Freshly isolated B cells were suspended in RPMI 1640 supplemented with 10% FCS, 100 U/ml penicillin, 100 µg/ml streptomycin, and 2 mmol/L l-glutamine (all from Sigma-Aldrich), and seeded at 5×10^5^ cells per well in 96-well U-bottom plate. Cells were either stimulated through CD40 alone (CD40 stimulation) or by sequential B-cell antigen receptor (BCR) engagement followed by CD40 signaling (dual BCR+CD40 stimulation) [5]. For the CD40 stimulation, B cells were stimulated for 48 hours with 1 µg/ml of soluble (s)CD40L (Enzo Life Sciences). For the dual BCR+CD40 stimulation, B cells were first incubated for 24 hours with goat anti-human IgG and IgM antibodies (Jackson ImmunoResearch) at 3.25 µg/ml, and then stimulated with sCD40L (1 µg/ml) for the next 48 hours. Secreted cytokine levels were quantified in culture supernatants using standard ELISA (BD Biosciences).

### Selection of candidate miRNAs for profiling

To narrow down the candidate miRNAs for our initial screen, we selected 102 miRNAs fulfilling either of the following 3 criteria: (i) miRNAs detected in at least 3 out of 4 recently published papers profiling miRNAs in resting human B cells [25]–[28], (ii) any miRNAs detected in human B cells and predicted to suppress LT, TNFα, or IL-10 by either of the following 3 target predicting software tools: TargetScanHuman (http://www.targetscan.org), miRDB (http://mirdb.org/miRDB), or miRanda (http://www.microrna.org); (iii) miRNAs previously implicated in any functional responses of B cells.

### RNA extraction and quantification of miRNA

Total RNA was extracted from cells using QIAzol (QIAGEN) and treated with DNaseI (QIAGEN) to digest any remaining genomic DNA in the samples. miRNAs were first quantified using a multiplex PCR system as described [29], [30], and then, for the implicated miRNA, findings were validated using TaqMan probe-based specific PCR assays (eg. Figure S2). Briefly, cDNAs for the 102 candidate miRNAs and RNU6B were synthesized from 200 ng of total RNA using Superscript III Reverse Transcriptase (Invitrogen) and mixture of stem-loop primers specific for each miRNA. The cDNA was amplified through 14 cycles of pre-PCR step. Quantitative PCR was performed on ABI Prism 7000 Sequence Detection System (Applied Biosystems) using SYBR Green PCR Master Mix (QIAGEN). Expression of each miRNA was normalized to the level of RNU6B, and the expression levels were calculated using ΔΔCT method. Primers used for reverse transcription and quantitative PCR are shown in Table S1.

### Quantification of mRNA and protein levels of sirtuin (SIRT)-1

The expression levels of SIRT1 mRNA were quantified by PCR, and were normalized to the level of glyceraldehyde 3-phophate dehydrogenase (GAPDH). The primer pair for SIRT1 was purchased from QIAGEN. DNA sequences for the primers used to quantify GAPDH are listed in Table S1. Protein levels of SIRT1 in isolated B cells were analyzed by Western blot using a monoclonal antibody against SIRT1 (abcam). Band density was analyzed using ImageJ software (http://rsbweb.nih.gov/ij/), and levels of SIRT1 were normalized to those of β-actin. B cell expression of SIRT1 protein was further assessed within total PBMC using flow cytometry. PBMC seeded at 3×10^5^ cells per well in 96-well U-bottom plate were kept unstimulated or stimulated sequentially with goat anti-human IgG and IgM antibodies (3.25 µg/ml) and sCD40L (0.5 µg/ml) for total of 72 hours. Harvested cells were stained for CD20 (clone H1; BD Biosciences) and were treated with Foxp3/Transcriptional factor staining buffer (Affymetrix ebioscience). Cells were then stained with anti-SIRT1 antibody (clone 19A7AB4) or with IgG isotype control (both from abcam). Specificity was confirmed using soluble recombinant SIRT1 which blocked the staining with anti-SIRT1 antibody (data not shown). Cells were analyzed on a LSR Fortessa flow cytometer (BD Biosciences) and the data were analyzed with FlowJo software (Tree Star). Dead cells were excluded using Live/Dead fixable cell stain (Life technologies).

### Manipulation of miRNA and SIRT1 activity in B cells

B cells (1×10^6^) were transfected with 200 nM of miR-132 mimic, or negative control RNA (NC: cel-miR-67) which has minimum sequence identity with human miRNAs (both from Dharmacon), using the Amaxa Human B cell Nucleofector Kit (Lonza) and Nucleofector I device (Lonza), according to their standard instructions. Dose-titration experiments were carried out to define optimal concentrations of the miR-132 mimic and the negative control. Transfected cells were suspended in 500 µl of medium in 48-well plates, and cultured for 72 hours with goat anti-human IgG and IgM antibody (3.25 µg/ml) together with 6×10^4^ of 60 Gy irradiated mouse fibroblast cell line (L cells), stably transfected with human CD40L (a gift from Dr Y. J. Liu, DNAX Research Institute of Molecular and Cellular Biology) [31].

For pharmacological manipulation of SIRT1, B cells were treated with either EX-527 (a selective inhibitor of SIRT1) or with resveratrol (a small molecule activator of SIRT1), at 10 µM (both from Sigma-Aldrich) for 48 hours, together with stimulation by sCD40L (1 µg/ml) and anti-human IgG and IgM (3.25 µg/ml), as described above.

## Results

### Abnormally increased effector cytokine responses are associated with elevated miR-132 expression in activated B cells of MS patients

**Figure 1:**
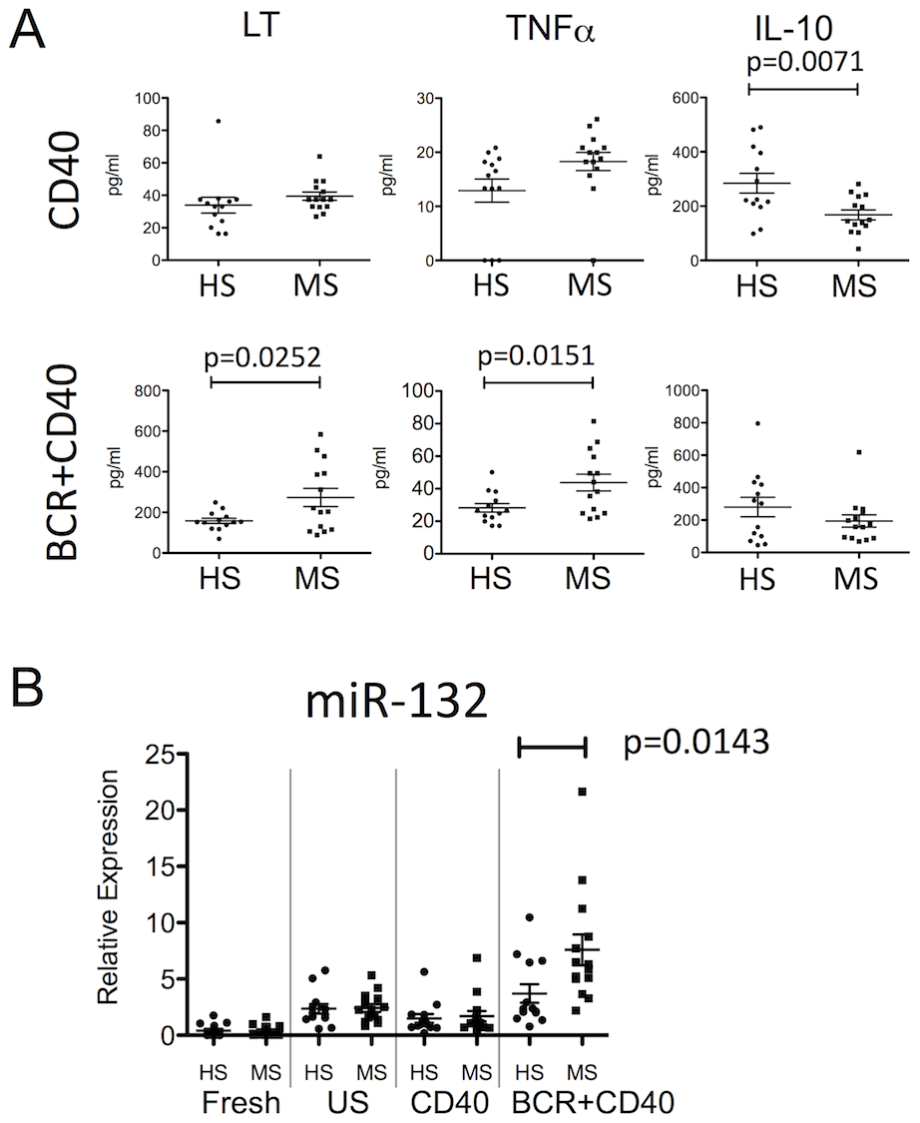
MS B cells express increased levels of miR-132, in association with an abnormal cytokine profile. A: Levels of lymphotoxin (LT), tumor necrosis factor (TNF)α, and interleukin (IL)-10 (CD40: following CD40 stimulation alone; BCR+CD40: following dual B-cell antigen receptor and CD40 stimulation) in the culture supernatants of B cells from healthy control subjects (HS, n = 13) and untreated MS patients (n = 14) (unpaired t-test). The proportion of memory cells among total B cells, and expression levels of CD40 were not different between B cells from MS patients and HS (data not shown). B: Expression level of miR-132 in B cells either immediately after isolation (Fresh), or following 48 hours in culture when left unstimulated (US), stimulated through CD40 alone (CD40), or stimulated through both the B-cell antigen receptor and CD40 (BCR+CD40). HS n = 13, MS n = 14; (Mann-Whitney U-test).

In order to dissect the molecular basis for the altered secretion of cytokines by MS B cells, we first compared cytokine profiles of activated B cells derived from untreated MS patients (n = 14; average age: 47.57±2.28; female:male = 12∶2) and HS (n = 13; average age: 47.23±2.68; female:male = 11∶2). B cells of MS patients exhibited an abnormal cytokine profile, involving significantly higher expressions of LT and TNFα upon ‘dual BCR+CD40 stimulation’, and significantly lower expression of IL-10 upon ‘CD40 stimulation’, as compared to HS B cells (Figure 1A), confirming our previously reported findings [5], [6]. To examine the potential association of the aberrant cytokine response of MS B cells with miRNAs within our pre-selected panel of 102 candidate miRNA, we used an initial screening step in a small cohort of patients and controls, followed by subsequent validation of implicated miRNAs in a larger cohort. Using multiplex qPCR, we screened for expression of the 102 candidate miRNAs within activated B cells of 5 MS patients and 5 age- and sex-matched HS. The screen (Table S2) suggested that expression levels of miR-132 (p = 0.0079) and miR-210 (p = 0.0362) were higher in MS B cells compared to HS B cells upon ‘dual BCR+CD40 stimulation’, and that miR-142-5p expression was more highly expressed in MS B cells upon ‘CD40 stimulation’ (p = 0.0317). We chose to focus on miR-132 as it was most strongly implicated in the screen and using the same PCR platform confirmed in a total cohort of 14 MS patients and 13 HS, that miR-132 expression was abnormally increased in ‘dual BCR+CD40 activated’ MS B cells (Figure 1B; p = 0.0143). This multiplex qPCR result was further validated using a separate dedicated qPCR system (Figure S2).

### Increasing miR-132 expression results in enhanced B cell production of LT and TNFα, and in reduced expression of SIRT1

**Figure 2:**
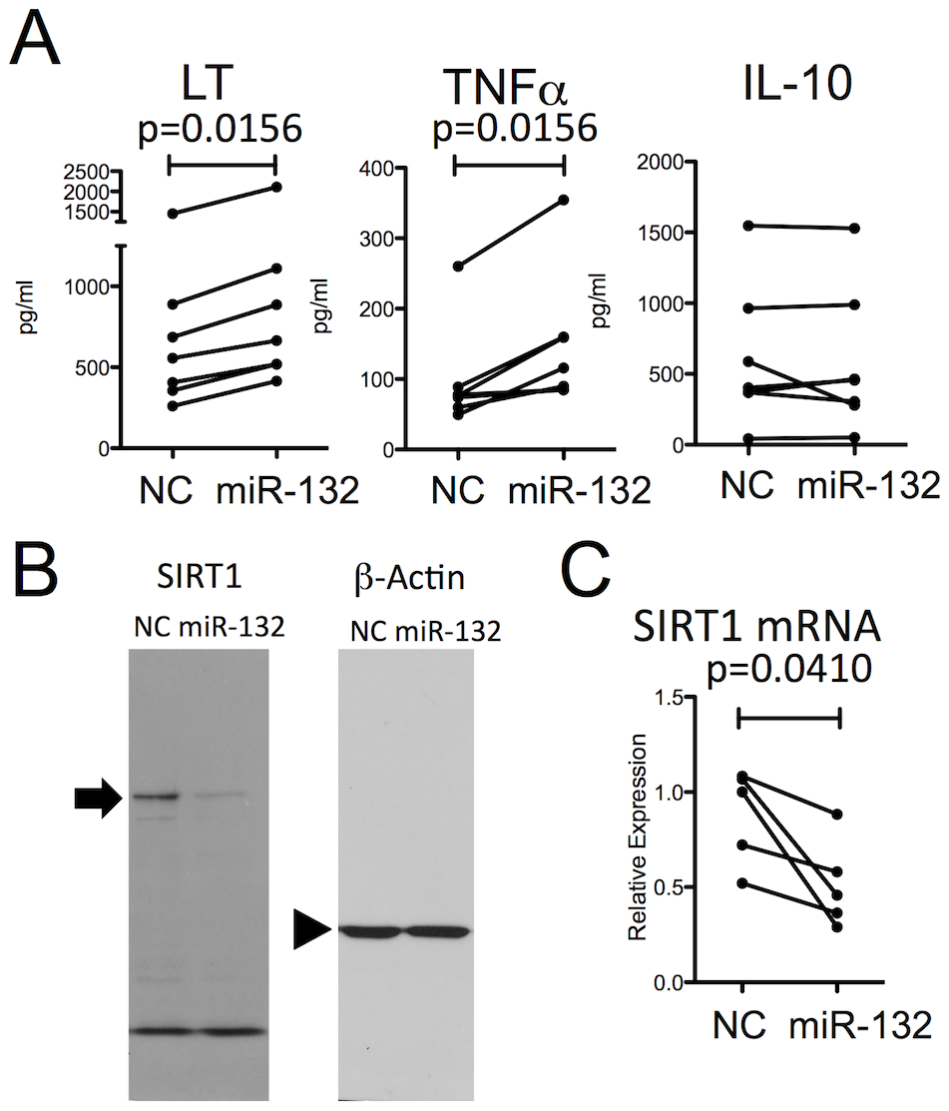
miR-132 enhances LT and TNFα production in association with SIRT1 suppression in B cells. A: Levels of lymphotoxin (LT), tumor necrosis factor (TNF)α, and interleukin (IL)-10 in B cells from healthy subjects (HS: n = 7) transfected with miR-132 mimic or negative control (NC), and stimulated through the B-cell antigen receptor (BCR) and CD40 (Wilcoxon test). B: Protein level of sirtuin (SIRT)-1 in B cells from HS transfected with miR-132 mimic or NC. A representative result of 2 experiments is shown. The arrow and the arrowhead indicate the bands corresponding to the molecular weight of SIRT1 and β-actin, respectively. C: Level of SIRT1 mRNA in B cells from HS transfected with miR-132 mimic or NC (n = 5) (Paired t-test).

Our observation that the higher secretion of LT and TNFα by MS B cells was associated with increased expression of miR-132 by the same B cells led us to hypothesize that these processes were mechanistically linked. To test this hypothesis, we overexpressed miR-132 (or a control miRNA) in HS B cells, and measured the impact of this manipulation on their cytokine production. Indeed, transfection of the miR-132 mimic into HS B cells resulted in significantly increased production of B cell LT and TNFα, without impacting IL-10 production (Figure 2A).

LT and TNFα were not predicted to be direct targets of miR-132 based on bioinformatics analyses, suggesting an indirect mechanism. To identify the molecular link between the abnormally elevated miR-132 levels in MS patient B cells and their enhanced LT and TNFα production, we considered molecules known to be suppressed by miR-132 [32]. Among these, SIRT1 appeared of particular interest because it had previously been implicated in T cell tolerance in mice [33]. We first used HEK293 cells and observed that transfection with the miR-132 mimic resulted in suppressed SIRT1 expression, both at the protein and mRNA levels (Figure S3). We next confirmed that miR-132 down regulates SIRT1 in primary human B cells through a similar over-expression approach (Figure 2B for protein; Figure 2C for mRNA).

### Activated B cells of MS patients express lower levels of SIRT1

**Figure 3:**
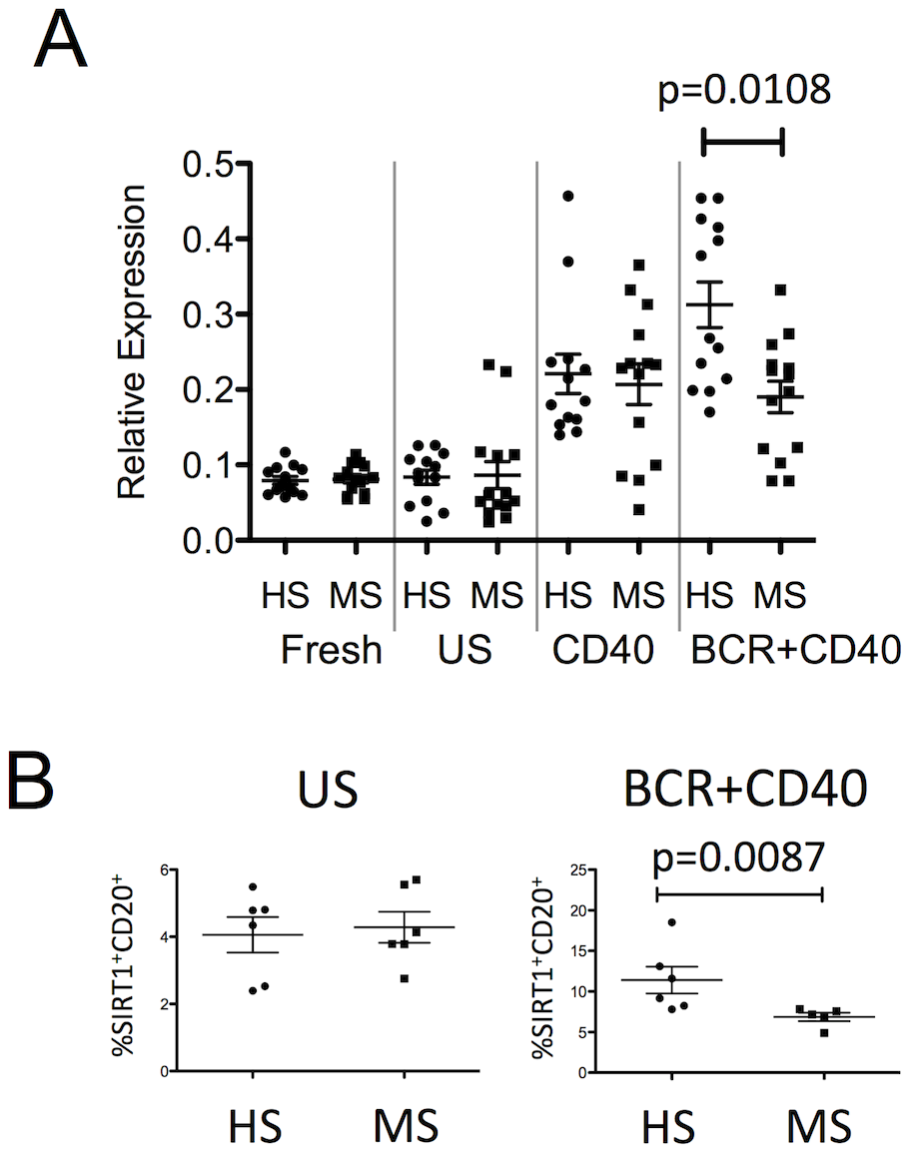
Activated B cells of MS patients express lower levels of SIRT1. A: Expression levels of SIRT1 mRNA in B cells immediately after isolation (Fresh), or when kept in culture for 48 hours either unstimulated (US), stimulated through CD40 (CD40), or stimulated through the BCR and CD40 (BCR+CD40) (Mann-Whitney U-test). B: Frequency of SIRT1^+^ B cells (CD20^+^) within whole PBMC that were kept unstimulated (US: left panel) or stimulated through the BCR and CD40 (BCR+CD40: right panel) (Mann-Whitney U-test).

We then returned to the clinical samples to assess the expression profile of SIRT1 and discovered that MS patient B cells exhibited significantly lesser induction of SIRT1 mRNA, compared with HS B cells, when activated with ‘dual BCR+CD40 stimulation’ (Figure 3A). In contrast, activation with ‘CD40 stimulation’ (which did not reveal differences in miR-132 expression between MS and HS B cells; Figure 1B), resulted in no differences in SIRT1 expression between MS and HS B cells. We also used flow cytometry to assess SIRT1 protein expression in PBMC samples of untreated MS patients (n = 5; average age: 41.20±5.76; female:male = 5∶0) and age-and-sex matched HS (n = 6; average age: 38.67±4.80; female:male = 6∶0). As shown in Figure 3B (right panel), activated B cells of MS patients exhibited lower SIRT1 expression, compared with B cells of HS (p = 0.0087).

### SIRT1 regulates abnormal LT and TNFα production by MS B cells

**Figure 4:**
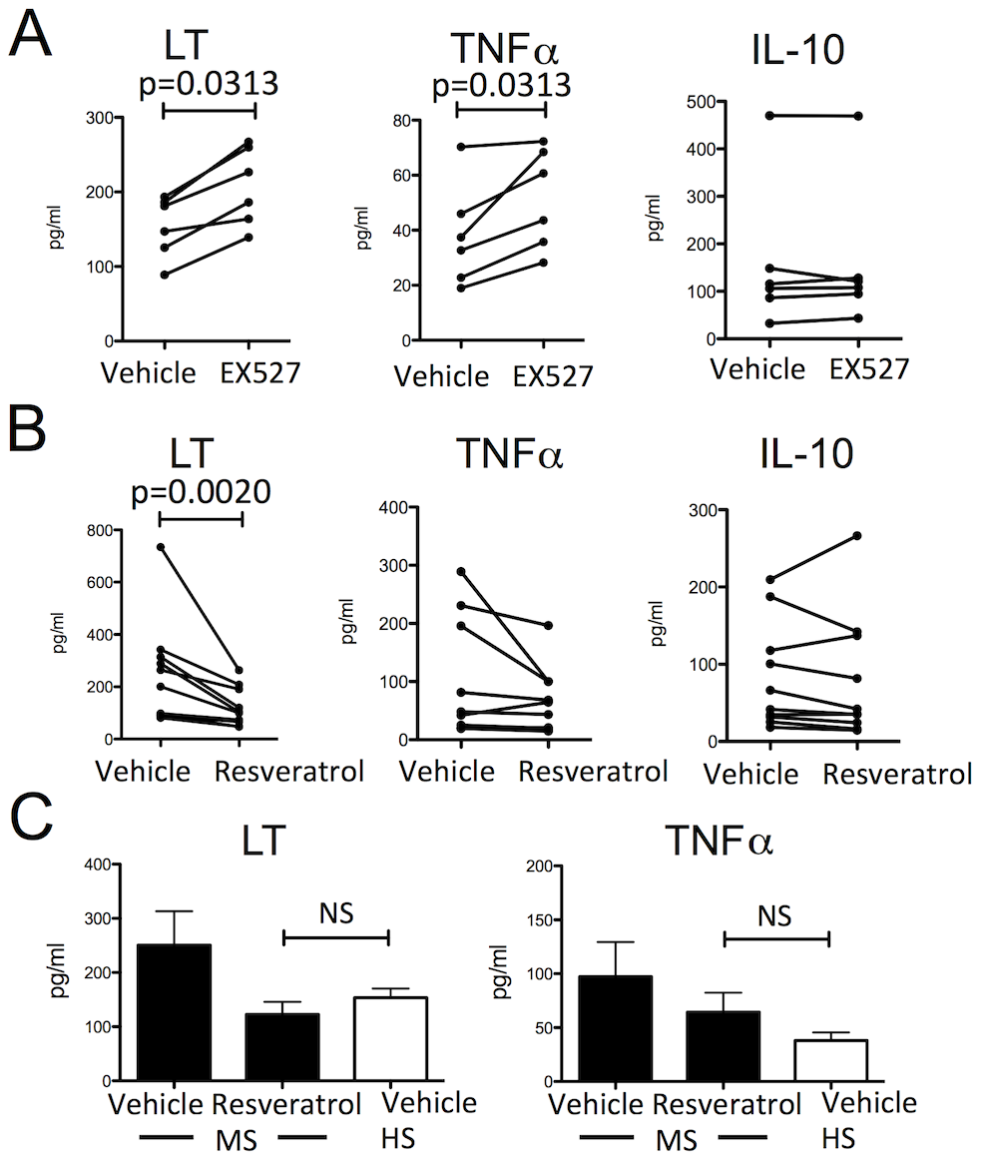
SIRT1 regulates LT and TNFα production from B cells. A: Levels of lymphotoxin (LT), tumor necrosis factor (TNF)α, and interleukin (IL)-10 produced by B cells from healthy subjects (HS: n = 6) treated with the selective pharmacological inhibitor of sirtuin (SIRT)-1, EX-527 (10 µM), or vehicle (0.1% DMSO), and stimulated through the B-cell antigen receptor and CD40 (Wilcoxon test). B: Levels of LT, TNFα, and IL-10 produced by B cells from MS patients (n = 10) treated with the small molecule activator of SIRT1, resveratrol (10 µM), or vehicle (0.1% DMSO), and stimulated through the B-cell antigen receptor and CD40 (Wilcoxon test). C: Levels of LT and TNFα produced by B cells from MS patients (n = 10) treated with either vehicle control (0.1% DMSO) or resveratrol (10 µM) and those from HS (n = 6) treated with vehicle control are shown. NS: not significant (unpaired t-test).

We next wished to directly examine whether SIRT1 was involved in the regulation of LT and TNFα by B cells. Treatment of HS B cells with EX-572, a selective pharmacological inhibitor of SIRT1, resulted in significantly enhanced B cell secretion of both LT and TNFα (Figure 4A), while secretion of IL-10 was not affected. Furthermore, resveratrol, a small molecule activator of SIRT1, significantly suppressed the abnormal production of LT by MS B cells (Figure 4B). TNFα production by MS B cells also appeared suppressed by resveratrol, particularly in patients who exhibited the highest levels of B cell TNFα production (Figure 4B), while IL-10 levels were not affected. Levels of LT secreted by MS B cells treated with resveratrol were similar to those secreted by vehicle treated HS B cells (Figure 4C), indicating that resveratrol treatment normalized the abnormal production of LT by MS B cells and appeared to partially but incompletely normalize the abnormal TNFα production.

## Discussion

The capacity of B cells to shape local immune responses through regulated expression of effector cytokines has emerged as an important antibody-independent function of B cells in both health and disease. In MS patients, abnormal expression of B-cell pro-inflammatory cytokines is thought to contribute to exaggerated effector T cell activation in the periphery leading to new disease relapses [6]. Our study provides novel insights into the molecular mechanisms underlying the aberrant pro-inflammatory cytokine responses of MS patient B cells. We demonstrate that, under certain contexts of activation, miR-132 expression is abnormally induced in B cells of MS patients and that such miR-132 induction results in over-production of the pro-inflammatory cytokines LT and TNFα. We further identify SIRT1 as a negative regulator of LT and TNFα, and implicate SIRT1 as the miR target responsible for the miR-132-mediated aberrant pro-inflammatory B cell cytokine responses observed in patients with MS.

To our knowledge, these findings represent the first description of miRNA regulation of B-cell cytokines. miR-132 was initially described as an important regulator of neuronal survival, maturation and differentiation and, more recently, implicated in several immune responses [32], [34]. Our results both extend the known list of normal miR-132 functions and implicate abnormally increased miR-132 expression in the exaggerated pro-inflammatory responses of MS patient B cells. Furthermore, our results indicate that suppression of SIRT1 by miR-132 contributes to the abnormally enhanced production of LT and TNFα by MS B cells. SIRT1, a member of the class III histone deacetylase family, has been implicated in a range of physiological and pathological processes including aging, metabolism, cancer and inflammation [35]. In the context of immune regulation, studies have shown a role for SIRT1 in suppressing T cell activity and impacting regulatory T cell function [33], [36]–[39]. A reduction of SIRT1 expression in PBMC of MS patients during relapse and its relation with T cell apoptosis was recently reported [39]. No study to our knowledge has thus far addressed a role of SIRT1 in B cells, though SIRT1-knockout mice spontaneously exhibit a lupus-like phenotype [33], [40]. The mechanism by which SIRT1 suppresses B cell LT and TNFα in our study remains to be elucidated, but may relate to its ability to suppress transcriptional activity of NF-κb [41] and AP-1 [33], which are known to be induced by BCR signaling and could facilitate LT and TNFα transcription.

We show here that inducing SIRT1 with reservatrol, could normalize the aberrant pro-inflammatory cytokine production of MS B cells. Targeting SIRT1 with reservatrol, a small molecule capable of accessing the CNS, may be a particularly interesting prospect in the context of MS. In addition to the role of B cells in augmenting T cell responses in the periphery of patients with MS, B cell-rich follicle-like structures have been described in the meninges of patients and the presence of these immune cell collections has been correlated with the extent of underlying subpial cortical injury that is now considered an important substrate of progressive MS [42]–[44]. This has led to the suggestion that abnormal MS B cell responses (including their aberrant production of LT and TNFα) within the meninges may contribute to propagating CNS-compartmentalized inflammation and the underlying cortical tissue injury. Targeting SIRT1 with a small molecule activator such as reservatrol may be beneficial in limiting pro-inflammatory B cell responses both in the periphery and in the target organ of patients with relapsing as well as progressive forms of the illness. In addition, activation of SIRT1 has also been reported to reduce neuronal damage in EAE [45], [46] and to mediate neuroprotection in animal models of Alzheimer's disease and amyotorophic lateral sclerosis [35]. Such neuroprotective activity, in addition to the B cell immune-modulatory potential, may make therapeutic targeting of SIRT1 a particularly attractive strategy for future MS therapy.

In summary, our findings indicate that the abnormally elevated levels of miR-132 which we discovered in B cells of MS patients, contribute to their aberrant expression of pro-inflammatory cytokines (LT and TNFα) and that the molecular mechanism involved in this B cell effector cytokine dysregulation in patients with MS involves miR-132-mediated suppression of SIRT1, which could provide an attractive therapeutic target for patients with MS.

## Supporting Information

Figure S1 **Purity of isolated B cells.** Isolated B cells were stained with fluorescein isothiocyanate (FITC)-anti-CD20, phycoerythrin (PE)-anti-CD3, or their isotype control antibodies (all from BD Biosciences), and analyzed on FACSCalibur flow cytometer (BD Biosciences) with FlowJo software (Tree Star).
(DOC)

Figure S2 **Expression level of miR-132 in activated B cells quantified by TaqMan quantitative PCR.** RNA extracted from B cells stimulated in through the B-cell antigen receptor and CD40 were reverse-transcribed into cDNA using TaqMan MicroRNA Reverse Transcription Kit (Applied Biosystems) with primers specific to miR-132 and RNU6B (both from Applied Biosystems), and their levels were quantified using TaqMan Universal PCR Master Mix (Applied Biosystems) on ABI Prism 7000 (Applied Biosystems). Expression of miR-132 was normalized to the level of RNU6B. **p<0.01 (Mann-Whitney U-test).
(DOC)

Figure S3 **Levels of SIRT1 protein and mRNA in HEK293 cells after miR-132 transfection.** HEK293 cells (American Type Culture Collection) were transfected with 37.5 nM of miR-132 mimic or negative control RNA (NC: cel-miR-67 which has minimum sequence identity with miRNAs in human) (both from Dharmacon) using Lipofectamin RNAiMAX (Invitrogen). Cells were collected after 48 hours, and protein and total RNA were extracted. A: Level of sirtuin (SIRT)-1 protein was quantified by Western blot. Representative band image for SIRT1 and β-Actin are shown (left). The arrow and the arrowhead indicate the bands corresponding to the molecular weight of SIRT1 and β-actin, respectively. Summary of 3 independent experiments are shown on the right. B: Levels of SIRT1 mRNA were quantified by qPCR. *p<0.05 (paired t-test).
(DOC)

Table S1 **DNA sequences of primers used for miRNA profiling.**
(XLSX)

Table S2 **Expression of the 102 candidate miRNA in activated B cells from HS and MS patients.**
(XLSX)
