# Correction: A Novel MicroRNA-132-Surtuin-1 Axis Underlies Aberrant B-cell Cytokine Regulation in Patients with Relapsing-Remitting Multiple Sclerosis

**DOI:** 10.1371/journal.pone.0109041

**Published:** 2014-09-23

**Authors:** The PLOS ONE Staff

There is an error in the title where Surtuin was misspelled. The correct title is: A Novel MicroRNA-132-Sirtuin-1 Axis Underlies Aberrant B-cell Cytokine Regulation in Patients with Relapsing-Remitting Multiple Sclerosis

The correct citation is: Miyazaki Y, Li R, Rezk A, Misirliyan H, Moore C, et al. (2014) A Novel MicroRNA-132-Sirtuin-1 Axis Underlies Aberrant B-cell Cytokine Regulation in Patients with Relapsing-Remitting Multiple Sclerosis. PLoS ONE 9(8): e105421. doi:10.1371/journal.pone.0105421
