# Supplementary material for: A Novel MicroRNA-132-Surtuin-1 Axis Underlies Aberrant B-cell Cytokine Regulation in Patients with Relapsing-Remitting Multiple Sclerosis
Source: PLoS One. 2014 Aug 19;9(8):e105421. doi: 10.1371/journal.pone.0105421 (PMC4138149; doi:10.1371/journal.pone.0105421)
Supplement: Figure S1 — Purity of isolated B cells. Isolated B cells were stained with fluorescein isothiocyanate (FITC)-anti-CD20, phycoerythrin (PE)-anti-CD3, or their isotype control antibodies (all from BD Biosciences), and analyzed on FACSCalibur flow cytometer (BD Biosciences) with FlowJo software (Tree Star). (DOC) [file pone.0105421.s001.doc]

**Figure S1: Purity of isolated B cells**

**
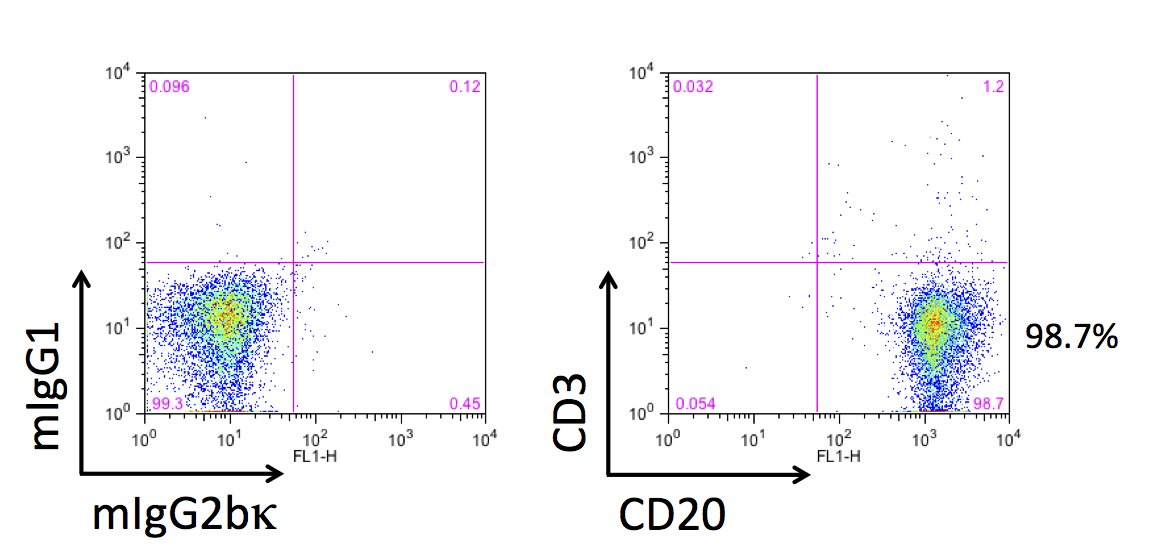
**

Isolated B cells were stained with fluorescein isothiocyanate (FITC)-anti-CD20, phycoerythrin (PE)-anti-CD3, or their isotype control antibodies (all from BD Biosciences), and analyzed on FACSCalibur flow cytometer (BD Biosciences) with FlowJo software (Tree Star).
