# Supplementary material for: A Novel MicroRNA-132-Surtuin-1 Axis Underlies Aberrant B-cell Cytokine Regulation in Patients with Relapsing-Remitting Multiple Sclerosis
Source: PLoS One. 2014 Aug 19;9(8):e105421. doi: 10.1371/journal.pone.0105421 (PMC4138149; doi:10.1371/journal.pone.0105421)
Supplement: Figure S2 — Expression level of miR-132 in activated B cells quantified by TaqMan quantitative PCR. RNA extracted from B cells stimulated in through the B-cell antigen receptor and CD40 were reverse-transcribed into cDNA using TaqMan MicroRNA Reverse Transcription Kit (Applied Biosystems) with primers specific to miR-132 and RNU6B (both from Applied Biosystems), and their levels were quantified using TaqMan Universal PCR Master Mix (Applied Biosystems) on ABI Prism 7000 (Applied Biosystems). Expression of miR-132 was normalized to the level of RNU6B. **p<0.01 (Mann-Whitney U-test). (DOC) [file pone.0105421.s002.doc]

**Figure S2: Expression level of miR-132 in B cells stimulated in X40 condition quantified by TaqMan quantitative PCR**

RNA extracted from B cells stimulated in the X40 condition were reverse-transcribed into cDNA using TaqMan MicroRNA Reverse Transcription Kit (Applied Biosystems) with primers specific to miR-132 and RNU6B (both from Applied Biosystems), and their levels were quantified using TaqMan Universal PCR Master Mix (Applied Biosystems) on ABI Prism 7000 (Applied Biosystems). Expression of miR-132 was normalized to the level of RNU6B. **p<0.01 (Mann-Whitney U-test)
