# Supplementary material for: A Novel MicroRNA-132-Surtuin-1 Axis Underlies Aberrant B-cell Cytokine Regulation in Patients with Relapsing-Remitting Multiple Sclerosis
Source: PLoS One. 2014 Aug 19;9(8):e105421. doi: 10.1371/journal.pone.0105421 (PMC4138149; doi:10.1371/journal.pone.0105421)
Supplement: Figure S3 — Levels of SIRT1 protein and mRNA in HEK293 cells after miR-132 transfection. HEK293 cells (American Type Culture Collection) were transfected with 37.5 nM of miR-132 mimic or negative control RNA (NC: cel-miR-67 which has minimum sequence identity with miRNAs in human) (both from Dharmacon) using Lipofectamin RNAiMAX (Invitrogen). Cells were collected after 48 hours, and protein and total RNA were extracted. A: Level of sirtuin (SIRT)-1 protein was quantified by Western blot. Representative band image for SIRT1 and β-Actin are shown (left). The arrow and the arrowhead indicate the bands corresponding to the molecular weight of SIRT1 and β-actin, respectively. Summary of 3 independent experiments are shown on the right. B: Levels of SIRT1 mRNA were quantified by qPCR. *p<0.05 (paired t-test). (DOC) [file pone.0105421.s003.doc]

**Figure S3: Levels of SIRT1 protein and mRNA in HEK293 cells after miR-132 transfection.**

HEK293 cells (American Type Culture Collection) were transfected with 37.5 nM of miR-132 mimic or negative control RNA (NC: cel-miR-67 which has minimum sequence identity with miRNAs in human) (both from Dharmacon) using Lipofectamin RNAiMAX (Invitrogen). Cells were collected after 48 hours, and protein and total RNA were extracted. A: Level of SIRT1 protein was quantified by Western blot. Representative band image for SIRT1 and β-Actin are shown (left). The arrow and the arrowhead indicate the bands corresponding to the molecular weight of SIRT1 and β-actin, respectively. Summary of 3 independent experiments are shown on the right. B: Levels of SIRT1 mRNA were quantified by qPCR. *p<0.05 (paired t-test)
